# Open Data Hackathon – Vergleich von in Präsenz und online durchgeführten Projektseminaren

**DOI:** 10.1365/s40702-021-00794-0

**Published:** 2021-09-27

**Authors:** Florian König, Daniel Wessel, Moreen Heine

**Affiliations:** Institut für Multimediale und Interaktive Systeme, Universität zu Lübeck, Lübeck, Deutschland

**Keywords:** Digitales Lernen, Hackathon, Open Data, Augmented Reality, Digital Learning, Hackathon, Open Data, Augmented Reality

## Abstract

Die Covid-19-Pandemie hat die Einführung und die Nutzung neuer, vollständig digitaler Lehrformate und -methoden in der Hochschullehre erforderlich gemacht. Insbesondere eher informelle, hoch-interaktive Lehrveranstaltungen wie Projektseminare müssen an diese veränderten Bedingungen angepasst werden. Aber wie können bei einer Online-Durchführung die Chancen des digitalen Formates ausgeschöpft werden und mögliche Probleme reduziert werden?

In diesem Beitrag kontrastieren wir die Durchführung eines Projektseminars in Form eines Hackathons in Präsenz mit einem Online-Format. Auf Basis des Stands der Forschung werden das Konzept, die Durchführung und die Ergebnisse dieser beiden Veranstaltungsformate verglichen. Der Fokus der Hackathons lag in beiden Fällen auf der Nutzung offener Daten im Kontext des öffentlichen Sektors (Open Data Hackathon). Auf Basis von qualitativen Teilnehmerbefragungen werden hierbei Verlauf, Projektergebnisse und Zufriedenheit der Teilnehmer verglichen. Dabei werden die unterschiedlichen Rahmenbedingungen (insbesondere bezüglich der Bedingungen für die Aufgabenbearbeitung und Zusammenarbeit) sowie Möglichkeiten zur Unterstützung der sozialen Interaktion untersucht und Vorschläge zur Durchführung von Online-Hackathons gegeben.

## Abstract (en)

The Covid 19 pandemic has necessitated introducing and using new, fully digital teaching formats and methods in higher education. In particular, more informal, highly interactive courses such as project seminars need to be adapted to the online format. How can the opportunities of digital learning formats be utilized, and potential problems be reduced?

In this paper, we contrast the implementation of a project seminar in the form of a hackathon in presence vs. online format. Based on the state of the art of research, we compare the concepts, their execution, and the results of these two modes. The thematic focus of the hackathons was the use of open data in the public sector (Open Data Hackathon). Based on qualitative participant surveys, the course, project results, and participants’ satisfaction are compared. The different conditions (especially regarding task execution and collaboration) and possibilities for supporting social interaction are examined, and suggestions for the implementation of online hackathons are provided.

## Einleitung

Projektseminare sind etablierte Lehrformate – besonders in gestaltungsorientierten Disziplinen. Ein besonderes Format sind dabei Hackathons, die auf einem klar begrenzten Zeitrahmen und kompakter Entwicklungsarbeit basieren. In Unternehmen haben sich Hackathons bereits vielfältig bewährt, auch um Externe im Rahmen von Open Innovation einzubeziehen. Im Vordergrund steht das Lösen von Problemen in begrenzter Zeit, Anforderungen an Perfektion werden zurückgestellt. Dieser Beitrag zeigt auf inwiefern das Format auch in der Lehre anwendbar ist – insbesondere vor dem Hintergrund der COVID-19-Pandemie. Im Fokus stehen Optionen und Erkenntnisse zur Gestaltung von virtuellen Hackathons. Zunächst werden Grundlagen zum Hackathon-Format sowie der Stand der Forschung präsentiert. Es folgt die vergleichende Vorstellung von zwei durchgeführten Lehrveranstaltungen (Präsenzveranstaltung im Wintersemester 2019/2020 und Online-Veranstaltung im Wintersemester 2020/2021) sowie das Vorgehen bei der Datenerhebung, die Ergebnisse der Teilnehmerbefragungen und deren Diskussion.

### Hackathons – konsequent ergebnisorientiert

Hackathons zeichnen sich durch eine effektive Problemlösung an einem Ort in begrenzter Zeit, die unmittelbare Abstimmung zwischen den Teammitgliedern, pragmatische Lösungsansätze für schnelle Erfolge, Experimentierfreude sowie explorative und spielerische Elemente aus (vgl. Schroll 2017). Betont werden außerdem Besonderheiten des Veranstaltungsortes: Dazu gehört die Verfügbarkeit von geschützten Zonen für die Gruppenarbeit, Reizen (z. B. spielerische Materialien je nach thematischer Ausstattung) und Ruhezonen. Zentral ist die Ermutigung, Neues auszuprobieren. Mit Hackathons sind außerdem spezifische Lernziele verbunden, insbesondere hinsichtlich der relevanten Technologien sowie zu Methoden, Kreativität, Führung, Kommunikation, Management und Präsentation (Briscoe und Mulligan 2015).

### Stand der Forschung

Projektbasiertes Lernen, auch in Form von Hackathons, wird in der Forschung in verschiedenen Fallstudien beschrieben. Dabei werden diverse Erfahrungen dargestellt und Empfehlungen abgeleitet, die für die Gestaltung solcher Formate relevant sind. Hierbei gehen Steglich et al. (2020) auf Faktoren ein, welche die Motivation zur Teilnahme beeinflussen können (z. B. Verbesserung der Teamwork-Fähigkeiten), beschreiben Erwartungen an den Hackathon (z. B. Arbeit in Teams) und die verwendeten Praktiken (z. B. Pair Programming). Besonders reizvoll sei die informelle Bildungsumgebung, um die technischen Fähigkeiten zu verbessern und ein Netzwerk mit Kommilitonen aufzubauen. In Gama (2019) wird von einem hohen Maß an Engagement und Begeisterung sowie hohem Lernerfolg im Rahmen eines Hackathons berichtet. Positiv sei außerdem die Wirkung von Hackathons hinsichtlich der Bindung der Studierenden an die Studienprogramme (Munro 2015) wie auch die beruflich relevante Vernetzung, wenn Praxispartner einbezogen werden (Porras et al. 2018).

Darüber hinaus werden jedoch auch negative Erscheinungen beschrieben. Porras et al. (2018) betrachten sowohl die Seite der Lehrenden (z. B. die nötige Vorbereitungszeit, die Notwendigkeit der Betreuung rund um die Uhr) als auch der Lernenden (insbesondere die hohe Belastung). Warner und Guo (2017) haben Faktoren erhoben, die Studierende von der Teilnahme an Hackathons abhalten bzw. die als negative Erfahrungen genannt wurden, darunter die körperliche Belastung, fehlende inhaltliche Substanz, ein zu wettbewerbsorientiertes Klima sowie Ängste, über nicht ausreichende Vorerfahrung zu verfügen.

Während das Thema virtueller Hackathons bisher in der wissenschaftlichen Literatur kaum beachtet wurde, fand es zu Anfang der COVID-19-Pandemie 2020 und 2021 vermehrt Beachtung. So kommt beispielsweise Bolten et al. (2021) zu dem Ergebnis, dass virtuelle Hackathons eine gute Alternative zur Kollaboration im Rahmen von Präsenzveranstaltungen sein können, wenn diese nicht möglich sind (z. B. aufgrund einer Pandemie oder räumlicher Distanz). Weitere Arbeiten adressieren öffentliche Hackathons, insbesondere zur Pandemiebekämpfung (zum Beispiel Temiz 2021). Der Fokus dieser Arbeiten liegt auf der Aktivierung von Freiwilligen und Organisationen sowie auf Fragen der Weiterentwicklung von Lösungsansätzen nach dem eigentlichen Hackathon.

Ziel dieses Beitrags ist, auf Basis einer vergleichenden Betrachtung Erkenntnisse zur erfolgreichen Umsetzung virtueller Hackathons im Kontext von Lehrveranstaltungen zu gewinnen, wobei die Erkenntnisse der bisherigen Forschung einbezogen werden. Da es sich bei den untersuchen Hackathons um Lehrveranstaltungen desselben Moduls handelt, konnten wir zwei Hackathons vergleichen, die sich bis auf die Präsenz bzw. Virtualität sehr ähnlich waren.

## Open Data Hackathon als Lehrveranstaltung in der Medieninformatik

### Zielgruppe und Qualifikationsziele

Der Open Data Hackathon wurde als Lehrveranstaltung im Masterstudiengang Medieninformatik konzipiert. Es sich hierbei um ein Wahlpflichtmodul, d. h. Studierende müssen aus einer vorgegebenen Liste von Modulen auswählen. Neben den Grundlagen der Medieninformatik aus dem entsprechenden Bachelorstudiengang wurden keine speziellen Module oder Vorwissen vorausgesetzt. Die Studierenden können sich unabhängig von ihrem aktuellen Fachsemester im Wintersemester in das Modul einschreiben.

Das Projektseminars beinhaltet klare Qualifikationsziele für die Teilnehmer. Dazu gehört ein Verständnis der Grundlagen zu Open Government, Open Data, Open Innovation und Data Driven Government. Insbesondere sollen Teilnehmer hierbei einen Einblick in Open-Data-Plattformen und -Anwendungen erlangen. Zusätzlich zu diesem fachlichen Wissen sollten Methoden und Werkzeuge kennengelernt werden und in der Zusammenarbeit (v. a. während der Softwareentwicklung) im Rahmen des Hackathons eingesetzt werden. Darüber hinaus sollen Teilnehmer die Gelegenheit bekommen, die Schlüsselqualifikation des Präsentierens – vor allem das Pitchen von Ideen – zu erproben und weiterzuentwickeln.

### Ablauf des Präsenz vs. Online-Hackathons

Der Ablauf der Open-Data-Hackathon-Lehrveranstaltung ist in Tab. 1 dargestellt. Bis auf das Hackathon-Wochenende war der grundlegende Ablauf in Präsenz und online identisch. Über das Semester verteilt fanden drei Veranstaltung statt, in denen das Konzept und formale Rahmenbedingungen sowie die inhaltlichen Grundlagen in das jeweilige Thema vorgestellt wurden. Durch diese vor- und nachbereitenden Sitzungen sollte die nötige inhaltliche Substanz sichergestellt werden (vgl. Warner und Guo 2017). Alle Teilnehmer haben ein eigenes Thema entwickelt und dieses an einem weiteren Termin im Rahmen eines Pitchs vorgestellt. Erst im Anschluss konnten sich die Teilnehmer zu Gruppen zusammenfinden und am Hackathon-Wochenende an einem gemeinsamen Thema arbeiten. Teilweise sind mehrere Ideen aus der Pitch-Phase in eine gemeinsame Projektidee eingeflossen. Das Hackathon-Wochenende wurde für den Online-Hackathon weiter an den Anfang des Wochenendes vorgezogen und die Bearbeitungszeit verkürzt, um eine Überlastung der Lehrenden und Teilnehmenden zu vermeiden (vgl. Porras et al. 2018).

**Tab. 1:** Ablauf des Open-Data Hackathons in Präsenz vs. Online

Vergleichskriterium	Präsenz	Online
Rahmenbedingungen	Termine in Präsenz Hackathon-Wochenende vor Ort in einem Innovation Lab	Durchführung komplett online via Webex
Ablauf	Einführung (3 Termine) Pitches (1 Termin)	
	Hackathon-Wochenende (Freitagabend bis Sonntagnachmittag)	Hackathon-Wochenende (Freitagmorgen bis Samstagmittag)
	Nachbesprechung (1 Termin)	
Thema	Verwendung und Aufbereitung von offenen Datenquellen. Dabei Lösung eines konkreten Problems oder Herstellung von Transparenz	Verwendung und Aufbereitung von offenen Datenquellen unter Verwendung von AR/VR sowie Einbeziehung rechtlicher Rahmenbedingungen
Sozialer Zusammenhalt während des Hackathons	Gemeinsamer Start und gemeinsamer Abschluss mit Ergebnisvorstellung	
	Alle Teilnehmer in einem großen Lab-Raum mit eigenen Arbeitsbereichen Gemeinsames Pizza-Essen	Gemeinsamer Webex Raum sowie separate Gruppenräume Care-Paket vorab mit gleichem Inhalt, Schlummertrunk, etc

Bei dem Präsenz-Hackathon fanden alle Sitzungen in den Räumlichkeiten der Universität statt und das Hackathon-Wochenende selbst in einem assoziierten Innovation Lab. In diesem konnte jeder Gruppe ein eigener Bereich zur Verfügung gestellt werden (abgetrennt mit mobilen Whiteboards und Schallschluck-Trennwänden). Diese boten den Gruppen einen Rückzugs- und Arbeitsbereich, erlaubten auf Wunsch aber auch den Austausch mit den anderen Gruppen. Um den sozialen Austausch anzuregen, wurde ein gemeinsames Pizza-Essen organisiert.

Aufgrund der COVID-19-Pandemie konnte der zweite Hackathon nicht in Präsenz durchgeführt werden. Alle Termine, einschließlich des Hackathon-Wochenendes, wurden stattdessen online über die Videokonferenzsoftware Webex Teams durchgeführt. Um möglichst viele Aspekte des Präsenz-Hackathons zu erhalten, wurde auf Webex ein gemeinsamer Raum für alle Teilnehmer eingerichtet und jeder Gruppe ein eigener Webex-Raum zur Verfügung gestellt (analog zu dem Raum bzw. Arbeitsbereich im Innovation Lab). Als Ersatz für das Pizza-Essen wurde ein Care-Paket zusammengestellt und im Vorfeld an die Teilnehmer geschickt. Es enthielt koffein-, vitamin- und energiereiche Komponenten und Material für Lab-Atmosphäre in den eigenen vier Wänden (z. B. Whiteboard-Folie und Whiteboard-Marker). Außerdem gab es gemeinsame Termine, wie ein gemeinsames Abendessen, einen Schlummertrunk um Mitternacht, und ein Exit-Game am nächsten Tag.

Für beide Hackathon-Lehrveranstaltungen war das übergeordnete Thema „Open Data“. Für den online durchgeführten Hackathon wurde zudem vorgegeben, für die Aufbereitung der offenen Daten Augmented Reality (AR) oder Virtual Reality (VR) zu nutzen, sowie die rechtlichen Rahmenbedingungen in die Lösung miteinzubeziehen.

### Bewertungskriterien

Der Open Data Hackathon umfasst einen Arbeitsaufwand von 4 Credit Points und wird als benotetes Modul angeboten. Die Leistungsbewertung deckt die Phasen im Hackathon ab – den individuellen Pitch, sowie als Teamleistung die Bearbeitung der Projektaufgabe (der eigentliche Hackathon) und die mediale Aufbereitung (Text‑/Bild- und ggf. Videomaterial für eine Webseite). Tab. 2 zeigt die Bewertungskriterien für die Aufgaben sowie deren Gewichtung für die Gesamtnote (in Prozent).

**Tab. 2:** Bewertungskriterien

Präsentation (Pitch) 10 %	Bearbeitung der Projektaufgabe 70 %	Mediale Aufbereitung 20 %
Klare Struktur Verständlichkeit Professioneller Vortragsstil Gestaltung der Visualisierung (z. B. Folien)	Relevanz des Problems Innovation Schöpfungshöhe (ausgehend vom Startpunkt) Praxisnähe Zukunftssicherheit/Weiterverwendbarkeit	Verständlichkeit des Texts Professionalität der Bilder/Animationen/Videos Dokumentation des Codes

## Themen und Ergebnisse

Gelenkt von dem Themenfeld (Open Data) sowie durch die spezifische Ausrichtung (Präsenz: konkretes Problem; Online: AR/VR in Kombination mit rechtlichen Rahmenbedingungen) wurden von den Teilnehmern unterschiedliche Ideen entwickelt. Tab. 3 und 4 zeigt die Themen, die in den individuellen Pitches vorgestellt wurden, und die letztlich umgesetzten Projekte. In den meisten Fällen wurden von den Teilnehmern Ideen kombiniert oder schon im Vorfeld abgestimmt. Im Präsenzformat ist dies häufiger erfolgt als im Online-Format.

**Tab. 3:** Vorgeschlagene und umgesetzte Themen (Präsenz)

Im Pitch vorgestelltes Thema		Im Hackathon bearbeitetes Thema
Hansetonne – Eine Web-Anwendung zur Ortung von Wertstoffinseln (vorgestellt durch zwei Teilnehmer)	→	Die Hansetonne – Eine Web-Anwendung zur Ortung von Wertstoffinseln
Nordratio – Offen Daten aus Norderstedt ins Verhältnis setzen und visuell aufbereiten	→	Nordratio – Wie können wir Open Data im Edutainment-Bereich nutzen?
Nordratio – Offen Daten aus Norderstedt automatisiert als Infografik auf einer Karte anzeigen	→	
Nordratio – Interaktive Aufbereitung der offenen Daten aus Norderstedt	→	
SH-Tycoon – Für die Stadt Kiel offene Daten spielerisch aufbereiten	→	SH-Tycoon Kiel – Wie kann Open Data über Edutainment vermittelt werden?
Die Ordnungshüter – Spielerisch Schleswig-Holstein kennenlernen	→	
Das nördlichste Quiz Deutschlands – Open Data für Schleswig-Holstein als Quiz aufbereiten	→	
Höher oder Tiefer? SH Edition – Schätzspiel basierend auf Open Data	→

**Tab. 4:** Vorgeschlagene und umgesetzte Themen (Online)

Im Pitch vorgestelltes Thema		Im Hackathon bearbeitetes Thema
Baltic Monuments – Denkmäler im virtuellen Raum erlebbar machen	→	VR Web-App Landmarks – Immersives Erkunden von Denkmälern in Augmented Reality
TILEZONE – Verteilung von regionalen Bodenflächen in Virtual Reality	X	
Visualisierung des Wasserverbrauchs von Haushalten in Schleswig-Holstein in Virtual Reality	X	
Datenvisualisierung mit Augmented Reality	→	AR Web App POLAR – Visualisierung der örtlichen Luftverschmutzung mittels Augmented Reality
PSA – Paragraphen Scanner App	X	
Luftqualität – Messwerte für Bürger sichtbar machen	→	
4‑Städte-Vergleich	X	Berlin Crime Run – Eine AR-App für mehr Freude an Bewegung durch das Lösen von Kriminalfällen
Open-Source Web-Plugin/API für die erfolgreiche Navigation	X	
Visualisierung/Auswertung Verkehrsdaten	X	

**Abb. 1:**
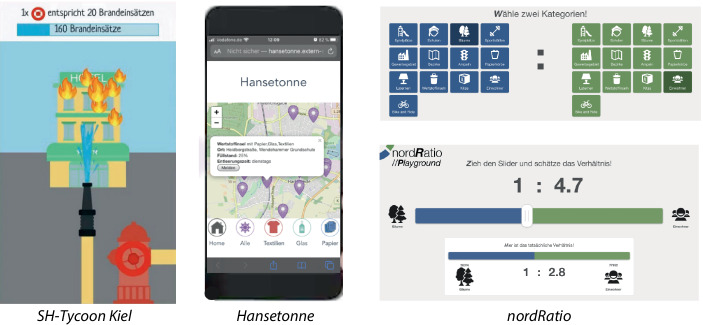
Ergebnisse Open Data Hackathon WS 2019 (Präsenz). *Links*: Eines von vier Spielen, in denen Open Data verwendet wird. *Mitte*: Eine Anwendung, welche die nächsten Wertstoffinseln (z. B. Papiercontainer) anzeigt. *Rechts*: Ein Schätzspiel, bei denen das Verhältnis von zwei Open-Data-Informationen geschätzt werden muss (z. B. Wie viele Bäume kommen auf jeden Einwohner?)

Abb. 1 und 2 zeigen die Ergebnisse der Hackathons mit kurzer Beschreibung. Die medialen Aufbereitungen der Ergebnisse mit tiefergehenden Beschreibungen und Abbildungen sind online zu finden unter https://jil.sh/open-data-hackathon und https://jil.sh/online-open-data-hackathon.

**Abb. 2:**
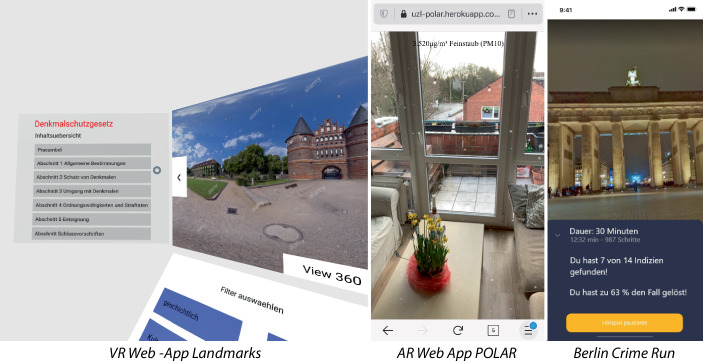
Ergebnisse Open Data Hackathon WS 2020 (Online). *Links*: Eine VR Visualisierung von Denkmälern der Stadt mit rechtlichen Aspekten. *Mitte*: Eine Anzeige der Feinstaubbelastung (als kleine Partikel) am aktuellen Ort basierend auf Open-Data-Informationen. *Rechts*: Ein Krimi-Spiel in Berlin

## Evaluation der Lehrveranstaltungen

Im Anschluss an die jeweilige Hackathon-Veranstaltung wurden die Teilnehmer gebeten, Feedback zur Lehrveranstaltung zu geben. Das Ziel der Evaluationen war es, positive und negative Aspekte im Konzept und in der Durchführung der Hackathon-Lehrveranstaltung zu identifizieren.

### Evaluationsdesign Präsenz

Die Evaluation des Präsenz-Hackathons erfolgte in Form eines semistrukturierten Interviews als Eins-zu-Eins-Gespräch in Präsenz. Der Interviewleitfaden bestand aus den drei Abschnitten „vor dem Hackathon“, „während des Hackathons“ und „abschließende Bewertung“. Der erste Abschnitt umfasst die bisherige Zusammenarbeit zwischen den Gruppenmitgliedern sowie die geplante Zusammenarbeit und Organisation in der Gruppe einschließlich verwendeter Methoden und Werkzeuge. Im zweiten Abschnitt wurde nach dem tatsächlichen Ablauf und der tatsächlichen Zusammenarbeit gefragt. Es ging hierbei um Abweichungen von der selbstgesetzten Planung, um die tatsächliche Koordination und Entscheidungsfindung sowie um kritische Ereignisse und Konflikte. Zudem sollten die Teilnehmer in diesem Abschnitt den Verlauf des Hackathons anhand der Parameter Müdigkeit, Stress und Spaß einschätzen. Im letzten Abschnitt des Interviews wurde erfragt, welches Wissen und welche Fertigkeiten den Teilnehmern persönlich geholfen haben bzw. geholfen hätten. Zudem sollten die Arbeitsbedingungen sowie der Hackathon insgesamt eingeschätzt werden und Aspekte, die gut waren und solche, die verbessert werden könnten, benannt werden. Abschließend bestand die Gelegenheit für weitere Anmerkungen und Punkte, nach denen nicht explizit gefragt wurde.

### Evaluationsdesign Online

Die Evaluation des Online-Hackathons erfolgte in Form eines Online-Fragebogens mit dem Umfragen-Tool LimeSurvey. Die Teilnehmer hatten in einem Zeitraum von zweieinhalb Wochen die Gelegenheit, an der Online-Befragung teilzunehmen. Zuerst wurden das Alter und Geschlecht sowie die Gruppe, in der sie im Hackathon gearbeitet hatten, erhoben. Der Fragebogen war wie der Interviewleitfaden thematisch in die drei Abschnitte „vor dem Hackathon“, „während des Hackathons“ und „abschließende Bewertung“ unterteilt. Im Abschnitt „vor dem Hackathon“ wurde gefragt, inwiefern Teilnehmer ihre Gruppenmitglieder bereits vor dem Hackathon kannten und inwiefern sie sich vorbereitet haben. Zudem wurde gefragt, wie die ersten zwei bis drei Stunden des Hackathons verbracht wurden, welche Werkzeuge verwendet wurden und welche Werkzeuge sich dabei als nützlich herausgestellt haben. Des Weiteren wurden die Teilnehmer gefragt, wie sie sich in ihrer Gruppe organisiert haben, wie mit Meinungsverschiedenheiten und wie mit technischen Schwierigkeiten umgegangen wurde. Wie bereits in den Interviews zum Präsenz-Hackathon sollten die Teilnehmer anschließend den Verlauf des Hackathons anhand der Parameter Müdigkeit, Stress und Spaß einschätzen und angeben, welches Wissen und welche Fertigkeiten ihnen persönlich geholfen haben und geholfen hätten. Zudem sollten die Arbeitsbedingungen sowie der Hackathon insgesamt eingeschätzt werden und der Hackathon in acht Kategorien mit einer Schulnote bewertet werden. Ebenfalls wie in den Interviews wurde abschließend gefragt welche Aspekte gut waren und welche verbessert werden könnten und Gelegenheit für weitere Anmerkungen gegeben.

### Ergebnisse Präsenz

Von den neun Teilnehmern der Lehrveranstaltung hatten sich sieben bereiterklärt, an den Interviews zur Evaluation teilzunehmen (3 weiblich, 4 männlich). Alle drei Gruppen des Hackathons waren dabei vertreten. Zwei Interviews wurden direkt am Ende des Hackathon-Wochenendes geführt. Alle weiteren Interviews wurden innerhalb von vier Wochen nach dem Wochenende geführt.

Da in den Interviews zum Teil viele Einzelnennungen getätigt wurden, wurde für die Auswertung eine Auswahl getroffen, basierend auf der Häufigkeit der Nennungen sowie der Relevanz für den Vergleich Präsenz- vs. Online-Veranstaltung. Die Ergebnisse sind in Tab. 5 dargestellt.

**Tab. 5:** Ausgewählte Ergebnisse Präsenz vs. Online

	Präsenz (*n* = 7)	Online (*n* = 6)
*Zusammenarbeit*		
Alle Gruppenmitglieder vorher bekannt	7/7	1/6
Vorherige Zusammenarbeit	5/7	x
Gruppenmitglieder nicht/flüchtig bekannt	x	3/6
Ein Gruppenmitglied bekannt	x	2/6
*Arbeitsorganisation*		
Vorarbeit	*5/7*	*5/6*
Erstellung Zeit- und Arbeitsplan	1/5	1/5
Aufgabenverteilung	4/5	x
Auswählen potenzieller Technologien	x	1/5
Vertraut machen mit Frameworks	x	1/5
Abspeicherung von Tutorial- & Hilfeseiten	x	1/5
Führung und Konfliktlösung		
Koordination im Team	5/7	6/6
Ohne Führungsrolle/eigenständige Koordination	4/5	x
Keine Durchsetzung von Entscheidungen gegen Widerstände	6/7	x
Keine schwerwiegenden Konflikte innerhalb der Gruppe	5/7	6/6
Entscheidung durch Verantwortlichen getroffen	2/7	x
Arbeitswerkzeuge		
Pair-Programming	1/7	2/6
Aufgabenliste am Whiteboard	3/7	x
Trello-Board	2/7	x
WhatsApp	1/7	x
ToDo-Liste	1/7	x
Discord	x	4/6
Visual Studio Code	x	2/6
GitLab bzw. Kanban Board	x	2/6
*Hilfreiche Fertigkeiten*		
Vorhandene Programmierkenntnisse	4/7	2/6
Vorerfahrungen aus Gruppenprojekten	4/7	x
*Fehlende Fertigkeiten*		
Defizite bei Programmierkenntnissen	5/7	4/6
*Arbeitsbedingungen*		
Gute Ausstattung der Arbeitsplätze	4/7	2/6
Gute Abschirmung der Gruppen	2/7	2/6
Wunsch nach besserer Home-Office Ausstattung	x	2/6
Wunsch nach besserem WLAN	x	2/6
Kommunikationsprobleme (abgehackte Verbindung, Mikrofon)	x	1/6
*Wünsche*		
Mehr Besprechungen/Rückmeldung	1/7	1/6
Mehr gemeinsame Mahlzeiten	2/7	x

*x* nicht genannt

Zusätzlich zu den in Tab. 5 genannten Punkten wurde als Probleme genannt, eine aufwändige Implementierung fehlerfrei umzusetzen bzw. spät abends mathematische Probleme zu lösen. Das Spielen von (für alle hörbare) Musik im Innovation Lab wurde z. T. negativ angemerkt.

Insgesamt war die Bewertung des Hackathons positiv, u. a. wurde genannt, dass der Hackathon weniger ernst gewesen sei, als erwartet, dass das Modul Spaß gemacht hat und die eigenen Erwartungen gut erfüllt wurden.

Auf die Frage nach abschließenden Anmerkungen gab es erneut eine Reihe von Einzelmeinungen. So wurde darauf hingewiesen, dass für den Hackathon eine gewisse Vorerfahrung notwendig sei und diese in den ersten Semestern eventuell nicht gegeben sei. Zudem wurde gesagt, dass es für die Arbeit hilfreich war, die anderen Gruppenmitglieder bereits in Vorfeld gekannt zu haben. Auch das gemeinsame Pizzaessen wurde positiv hervorgeheben.

### Ergebnisse Online

Von den neun Teilnehmern der Lehrveranstaltung haben sechs an der Online-Umfrage teilgenommen (zwei weiblich, vier männlich). Hierbei wurden nur vollständig bearbeitete Umfragen einbezogen. Mit den sechs Teilnehmern waren alle drei Gruppen des Hackathons in der Umfrage vertreten. Die Umfrage wurde knapp zwei Monate nach dem Hackathon freigeschaltet und die Teilnehmer hatten dann für zweieinhalb Wochen die Gelegenheit, an der Online-Befragung teilzunehmen. Die Ergebnisse sind ebenfalls in Tab. 5 dargestellt.

Zusätzlich zu den in Tab. 5 genannten Punkten sahen die Teilnehmer Defizite bei der Vorbereitung darin, dass sie (1) im Hackathon nicht die geplante Technologie verwendet haben, (2) sich nicht gut genug überlegt haben, „wo die Reise hin gehen soll“, (3) die Arbeit mit den geplanten Frameworks nicht ausreichend geübt hatten und (4) die aufgetretenen Probleme im Vorfeld nicht hinreichend antizipiert haben. Bezüglich der Aufgabenverteilung gaben mehrere Teilnehmer an, die Aufgaben entsprechend den Kenntnissen und Fähigkeiten der Gruppenmitglieder verteilt zu haben. Zwar wurde mehrfach angegeben, dass Entscheidungen gemeinsam getroffen wurden, doch scheint es zumindest in einem Teil der Gruppen Expertenrollen für bestimmte Themen gegeben zu haben, deren Stimme im Zweifel ein höheres Gewicht beigemessen wurde. Ursache für Meinungsverschiedenheiten waren häufig die Priorisierung beziehungsweise der Sinn einzelner Features.

Die Teilnehmer wurden gebeten, ein kritisches Ereignis sowie dessen Lösung zu beschreiben. Es wurden sehr spezifische Probleme bei der Programmierung oder der Einrichtung der Entwicklungsumgebungen genannt. Dabei wurde auf pragmatische Lösungen gesetzt und der Behebung kleinerer Bugs eine geringe Priorität gegeben. Als weiteres Problem wurde Zeitdruck beim Bereitstellen der entwickelten Anwendung kurz vor dem Ende des Hackathons genannt. Auch wurde gewünscht, im Vorfeld noch genauer vermittelt zu bekommen, worauf es im Hackathon ankommt, was als Produkt des Hackathon erwartet wird (inkl. Verwendung von GitLab zur Code-Verwaltung), und inwieweit während der Veranstaltung vom Plan abgewichen werden kann, ohne Abzüge bei der Benotung zu riskieren.

Als weitere Anmerkungen am Ende der Online-Umfrage wurde von einem Teilnehmer angemerkt, dass das Thema mit den drei Vorgaben (1) Open Data, (2) AR oder VR und (3) rechtliche Aspekte zu stark eingeschränkt gewesen sei. Als Vorschlag wurde genannt, dass ein Teil der Anforderungen als Alternativen angeboten werden könnten.

Die Teilnehmer haben zudem beantwortet, was ihnen besonders in Erinnerung geblieben ist. Die Antworten lassen sich in zwei Kategorien einteilen: Zum einen das Rahmenprogramm zusammen mit der Interaktion mit den anderen Teilnehmern des Hackathons, zum anderen das konzentrierte Arbeiten am eigenen Projekt sowohl mit Erfolgserlebnissen als auch mit Frustration. Zweimal wurde das Care-Paket mit kleinen Snacks genannt, das den Teilnehmern zur Verfügung gestellt wurde. Zudem eine lockere und sympathische Stimmung und die Begegnung untereinander auf Augenhöhe. Auch Auflockerungen, wie Memes im Webex-Chat und die Events zwischen den Programmierphasen wurden positiv hervorgehoben. In Bezug auf die Arbeit am Projekt wurde die Möglichkeit, mehrere Stunden am Stück „durchzucoden“, ein Verlust des Zeitgefühls sowie das Annehmen neuer Herausforderungen genannt. Auch eher negative Aspekte, wie Demotivation bei nicht funktionierendem Programmcode oder umgeworfenen Konzepten sind den Teilnehmern in Erinnerung geblieben.

### Diskussion

Eine vergleichende Betrachtung der Bewertungen des Präsenz-Hackathons und des Online-Hackathons ergibt die folgenden Gemeinsamkeiten. Als informelle Lehrveranstaltung, die benotet in den Masterabschluss eingeht, besteht auf Seiten der Teilnehmer ein berechtigtes Interesse nach klaren Informationen bezüglich der Anforderungen und Notengebung. Zwar wurden Informationen über die Bewertung geben (siehe Tab. 2), allerdings war offenbar nicht allen Teilnehmern klar, wie genau sie sich an den zuvor angekündigten Plan halten müssen. Möglicherweise hat die Sorge vor Punktabzügen die Teilnehmer hier in ihrer kreativen Freiheit beschränkt. Dieses Thema sollte zukünftig offen angesprochen und die Teilnehmer zum Ausprobieren ermuntert werden.

Zwar wurden den Teilnehmern sowohl in Präsenz als auch online während des Hackathon-Wochenendes Gespräche zum Austausch mit den Dozierenden angeboten und mindestens ein Dozent war während der gesamten Zeit erreichbar, doch zeigt das Feedback, dass hier weitere explizite Angebote hilfreich sein könnten (z. B. durch ein Farbsystem im Sinne von „grün = alles in Ordnung“, „gelb = Feedback gewünscht“, im Raum via Karten, online via Profilbilder der WebEx Räume).

Bei beiden Terminen sind die vorgegebenen Pausen und Events zwischen der eigentlichen Programmierphasen in positiver Erinnerung geblieben. Soziale Ereignisse – in Präsenz durch ein gemeinsames Pizza-Essen, online durch Essen von gleichen Snacks (aus den Care-Paketen) – sollten daher beibehalten werden. Der Hackathon als eher informelle Lehrveranstaltung lebt von diesen sozialen Interaktionen, die sowohl in Präsenz als auch Online gut angeregt werden können.

Mehrere Teilnehmer wünschten sich eine Einführung in die Programmierung oder gaben an, hier individuelle Defizite festgestellt zu haben. Zwar wurde im Vorfeld des Online-Hackathon eine einführende Veranstaltung zu AR/VR angeboten, doch scheint hier noch zusätzlicher Bedarf an einer eher praktisch orientierten Einführung zu bestehen. Es sollte daher erwogen werden, dies noch mehr in den Einführungsveranstaltungen im Vorfeld des Hackathons zu thematisieren.

Den Teilnehmern sollten vor zukünftigen Hackathons mehr Information gegeben werden, mit welcher technischen Ausstattung sie rechnen können und welche Software-Werkzeuge gestellt werden und gegebenenfalls vorgegeben sind. Auch könnte der Arbeitsprozess der Teilnehmer weiter unterstützt werden, indem ein Austausch zwischen den Gruppen aktiv gefördert wird.

Unterschiede zwischen dem Präsenz- und Online-Hackathon waren eher organisatorischer Art. Der Wechsel der Anfangszeit des Hackathons von Freitagabend auf Freitagvormittag führte zu Kollisionen mit anderen Lehrveranstaltungen. Zwar hatten die Teilnehmer nicht auf dieses Problem hingewiesen, doch sollten derartige Terminkonflikte zukünftig frühzeitig abgefragt werden. Auch ein Wechsel zurück auf den Freitagabend sollte erwogen werden, wobei hier eine stärkere Belastung der Teilnehmer zu erwarten ist (zwei Nächte). Eine Verkürzung des Hackathons ist hier denkbar.

In der Präsenzveranstaltung kannten sich im Vergleich zum Online-Hackathon mehr Teilnehmer bereits vor dem Hackathon. Möglicherweise hatte hier die generell stattfindende Online-Lehre Einfluss, insbesondere für Teilnehmer, die nicht zuvor an der gleichen Universität (im Bachelor) studiert hatten. Die Arbeit allein zu Hause im Rahmen des Online-Hackathons wurde von den Teilnehmern zum Teil explizit als Vorteil (z. B. ungestörtes Arbeiten), teilweise jedoch auch (z. B. aufgrund unzureichender Ausstattung) als Hindernis wahrgenommen. Für zukünftig online stattfindende Hackathons sollte daher noch stärker darauf geachtet werden, die Vorteile dieses Formats zu nutzen und mögliche Nachteile noch besser abzufangen (z. B. durch Bereitstellung von Leihgeräten oder universitären Einzelpersonen-Arbeitsräumen).

## Fazit

Ein Hackathon ist eine hoch-interaktive, informell geprägte Lerngelegenheit, die den Teilnehmern die Möglichkeit gibt, ihre Fertigkeiten zu einem konkreten Thema mit begrenzter Zeit unter Beweis zu stellen. Auch wenn die Bedingungen für einen Präsenz-Hackathon einige Vorteile bietet – u. a. einfachere, spontanere soziale Interaktion – kann das Format des Hackathons mit wenigen Anpassungen auf ein Online-Format übertragen werden. Bei Betrachtung der entstandenen Produkte sind Online-Hackathons durchaus konkurrenzfähig mit Präsenz-Hackathons.

Trotzdem sind Zweifel angebracht, inwieweit Online-Hackathons präferiert werden sollten. Zwar können soziale Prozesse unterstützt werden, jedoch führt die gemeinsame Arbeit vor Ort zu Gelegenheiten der Kommunikation und Interaktion, die nur schwer mit Online-Tools repliziert werden können. Während sich Lernziele und Aufgabenstellung sehr gut übertragen lassen, profitiert die soziale Interaktion von Unterstützungsmaßnahmen. Im konkreten Fall wurde mit Care-Paketen eine Verbundenheit hergestellt, da alle die gleichen Drinks, Snacks und Arbeitsmaterialien hatten. Auch gemeinsame Pausenzeiten wurden sehr positiv aufgenommen. Hackathon-Formate können erfolgreich virtuell durchgeführt werden, mehr Gelegenheit zum direkten Austausch wird jedoch in Präsenzformaten geboten. Dieser direkte Austausch und die Vernetzung im Rahmen eines eher informellen Lernformats sind für die Teilnehmer besonders wertvoll.
